# Indole-3-Propionic Acid, a Gut Microbiota Metabolite, Protects Against the Development of Postoperative Delirium

**DOI:** 10.1097/SLA.0000000000005886

**Published:** 2023-04-27

**Authors:** Xue Zhou, Xinbo Wu, Yan Wu, Liuyue Yang, Eleanor Shi, Weihua Ding, Liang Chen, Xu Shi, Xia Feng, Chienwen Su, Zerong You, Jianguo Xia, Cynthia Chen, Vladimir Yeliseyev, Lynn Bry, Suyun Xia, Peigen Huang, Jiawei Meng, Timothy Houle, Oluwaseun Akeju, Jianren Mao, Robert Gerszten, Qian Chen, Zhongcong Xie, Shiqian Shen

**Affiliations:** * Department of Anesthesia, Critical Care and Pain Medicine, Massachusetts General Hospital, Harvard Medical School, Boston, MA; † Department of Orthopedics, Shanghai Tenth Hospital, Tongji University School of Medicine, Shanghai; ‡ Department of Anesthesiology, the First Affiliated Hospital of Sun Yat-sen University, Guangzhou, China; § Dana Farber Cancer Institute, Harvard Medical School, Boston, MA; ∥ Center for Discovery and Innovation, Hackensack Health Care, Nutley, NJ; ¶ Department of Cardiovascular Medicine, Beth Israel Deaconess Medical Center; # Department of Pediatrics, Massachusetts General Hospital, Harvard Medical School, Boston, MA; ** Department of Parasitology, McGill University, Montreal, Canada; †† Department of Pathology, Brigham and Women’s Hospital; ‡‡ The Steele Lab, Department of Radiation Oncology, Massachusetts General Hospital, Harvard Medical School, Boston; §§ McGovern Institute for Brain Research, Massachusetts Institute of Technology, Cambridge, MA; ∥∥ Shanghai Institute of Materia Medica, Chinese Academy of Sciences, Shanghai, China

**Keywords:** gut microbiota, indole-3-propionic acid, anesthesia/surgery, postoperative delirium

## Abstract

**Objective:**

The aim was to determine preoperative gut microbiota metabolites that may be associated with postoperative delirium (POD) development in patients and further study in rodents.

**Summary Background Data:**

POD occurs in 9% to 50% of older patients undergoing anesthesia/surgery but lacks effective treatments or prevention. High-throughput metabolomics using liquid chromatography with tandem mass spectrometry has accelerated disease-related biomarkers discovery. We performed metabolomic studies in humans to identify potential metabolite biomarkers linked to POD and examined potential mechanisms in rodents.

**Methods:**

We performed a prospective observational cohort study to examine the metabolomic changes that were associated with the development of POD. Then the gut microbiota-related metabolomic changes were recapitulated by gut microbiota perturbation in rodents. POD was assessed in mice using a battery of behavioral tests including novel objective test, Y-maze test, open-field test, and buried food test. The mechanisms through which gut microbiota-related metabolomic changes influenced POD were examined using chemogenetics.

**Results:**

Indole-3-propionic acid (IPA) is a gut microbiota metabolite that belongs to the indole family. Baseline plasma levels of IPA were significantly inversely correlated with the onset of POD in 103 (17 cases) human individuals. This relationship was validated in preclinical mouse models for POD: reducing IPA levels through gut microbiota perturbation promoted POD-like behavior. More importantly, IPA administration deterred POD-like behavior. Colonization of germ-free mice with mutant *Clostridium sporogenes* that did not produce IPA-promoted POD-like behavior. Chemogenetic studies revealed that the protective effect of IPA in mice was mediated, in part, by peroxisome proliferator-activated receptor gamma coactivator 1-alpha in hippocampal interneurons.

**Conclusions:**

Gut microbiota-derived IPA is an important molecule implicated in the pathogenesis of POD, which could potentially be harnessed for POD prevention.

Postoperative delirium (POD) occurs in 9% to 50% of older patients undergoing anesthesia/surgery, and is associated with greater incidences of postoperative complications, worse clinical outcomes, higher mortality, and development of Alzheimer’s disease (AD).^1^ With population aging and an increasing demand for anesthesia/surgery, POD has become a pressing issue with great public health relevance. The pathogenesis of POD remains largely unclear. Age and preoperative cognitive impairment are associated with the development of POD.^2^ More recently, mitochondrial dysfunction has been linked to the onset of POD.^3,4^

Gut microbiota is the consortium of microorganisms residing in the gastrointestinal tract. It has been increasingly recognized as an important player in host health and disease. For example, gut microbiota is linked to gastrointestinal homeostasis and inflammatory bowel diseases. More interestingly, gut microbiota appears to modulate neuroimmune interactions and has been associated with AD, Parkinson’s disease, multiple sclerosis, and pain.^5–8^ Accumulating evidence supports a role of gut microbiota in POD.^9^ In rodents, gut microbiota dysbiosis was shown to contribute to the pathogenesis of POD that was attenuated by a probiotic treatment.^10^ In orthopedic patients, surgery exacerbated both preexisting microbiome dysbiosis and intestinal barrier dysfunction in patients with prodromal AS. Importantly, these changes might be associated with systemic inflammation resulting in further cognitive decline.^11^

Despite the increased awareness of gut microbiota in POD, it remains largely unknown the metabolomic changes related to gut microbiota and how they might contribute to POD. Recently, metabolomics has been widely used to identify novel biomarkers and generate mechanistic insights into many diseases, including metabolic, cardiovascular, and renal diseases.^12–14^ In the current study, we performed metabolomics in a prospective patient cohort and identified gut microbiota-related metabolomic changes implicated in POD. A gut microbiota-related molecule was then examined in a preclinical animal model for its potential to prevent POD and plausible mechanisms of action.

## MATERIALS AND METHODS

### Human Subjects

We performed a prospective observational cohort study (IRB 2006P001288) at the Massachusetts General Hospital between 2016 and 2020 in patients (65 y or older and were proficient in English) scheduled to have an elective knee replacement, hip replacement, or laminectomy at the study hospital.^15^ The exclusion criteria included (1) past medical history of neurological and psychiatric diseases including AD, other forms of dementia, stroke, or psychosis; (2) severe visual or hearing impairment; (3) were current smokers; or (4) taking antibiotics within 1 week of the day of surgery because disturbance of gut microbiota may confound data interpretation. We obtained written informed consent at the time of enrollment. All participants received standardized perioperative care determined by their clinical treatment teams. Trained clinical research coordinators performed Confusion Assessment of Measurement (CAM)^16,17^ in the participants on postoperative day 1 and/or 2 between 8:00 am and 12:00 noon to assess POD. Study participants who were diagnosed of POD for at least 1 of the 2 days were in the POD group, those who did not meet diagnosis for POD on either postoperative days were in the No-POD group. Besides POD assessment, demographic information for study participants was also recorded. Human assessment was performed by study staff who was blinded to study design. A total of 103 patients whose samples were available for analyses were included. For power analysis, using 2 groups, 2-sided equality, group 1 mean of 0.125, and group 2 mean of 0.1, standard deviation of 0.025, sampling ratio of 5, type I error rate of 0.05, for power of 0.8, 60 patients are needed; for power of 0.9, 78 patients are needed.

#### Human blood sample

Blood samples were obtained before anesthesia/surgery by phlebotomists or certified clinicians. Blood samples were collected in EDTA tubes followed by centrifugation of 2000g for 10 minutes at 4°C. The resulting supernatant (plasma) was immediately transferred to clean microcentrifuge tubes and stored at −80°C. Multiple free-thaw cycles were avoided.

#### Blinding

Blood samples were labeled with numbers and were analyzed by chemists who were blinded to the study design. Human CAM assessors did not participate in blood samples collection or analysis. Blood metabolomics were analyzed before clinical POD data were made available.

### Mice

All procedures and animal use were approved by the MassGeneral Brigham Institutional Animal Care and Use Committee and were in accordance with the guidelines established by National Institutes of Health and the International Association for the Study of Pain. Female C57BL/6 mice were purchased from the Jackson Laboratory (Bar Harbor, ME). Mice were used at 4 to 6 months of age. Randomization for group assignment was performed where appropriate. For gut microbiota perturbation, mice were provided with 0.5 g/L Ampicillin in drinking water supplied ad libitum starting 2 weeks before anesthesia/surgery and was maintained through the entire experimental period, with water changed every other day. For germ-free mice, female C57BL/6 mice (4 to 6 mo old) were used for colonization. Specifically, mice were colonized with either wild-type *Clostridium sporogenes* (ATCC strain 15579) or *fldC* mutant *C. sporogenes* by oral gavage (200 μL, ∼1 × 10^7^ CFU), once weekly for 2 times. Fecal culture and polymerase chain reaction (PCR) were performed to confirm successful monocolonization. Indole-3-propionic acid (IPA) intraperitoneal injection: IPA was dissolved in 0.9% NaCl:ethanol (v/v 10:1), and injected twice daily at 0.0625 mmol/kg.

### Surgery

Mice were anesthetized with isoflurane vaporizer. Lidocaine 1% plain was used for skin infiltration. Surgeries were performed in bilateral thigh to expose femoral artery with care taken not to dissect femoral/sciatic nerve. Skin incision was about 0.6 cm each side; wound was closed with 2-0 silk suture. For Sham procedure, animals underwent isoflurane anesthesia for similar duration. No incision was made. For immediate postoperative pain control, skin was infiltrated with 0.25% bupivacaine and wound was covered with EMLA cream (AstraZeneca, Wilmington, DE). US). The hindpaw mechanical withdraw threshold, a widely accepted assessment tool for lower extremity injuries,^18^ was used to assess pain-like behavior in mice and the effect of pain control.

### Gut Microbiota Shot-gun Sequencing

Fecal samples were collected for DNA extraction using Qiagen PowerFecal DNA kit (Qiagen, Valencia, CA, USA) according to the manufacturer’s instruction. DNA samples were sent to CD Genomics (Long Island, NY) for shot-gun sequencing using Illumina NovoSeq. Raw reads were quality-filtered using KneadData version 0.8.0 (http://huttenhower.sph.harvard.edu/kneaddata). Quality-filtered reads were mapped against the mouse genome (mouse_C57BL_6NJ) using Bowtie 2 v2.4.1 to remove reads from host genome.^19^ Metagenomes were then taxonomically profiled using MetaPhlAn2 v2.8.1 using default parameters.^20^ Functional profiling was performed using HUManN2 v2.8.1 with UniRef90 mode.^21^ The unstratified gene-level abundances were converted to both gene ontology (GO) terms and kyoto encyclopedia of genes and genomes orthologs. The gene family and pathway abundance results were further normalized to relative abundance and regrouped according to GO terms and kyoto encyclopedia of genes and genomes orthologs. In addition, Knead data-processed reads were classified using Kraken2 v2.1.0 to assign taxonomy,^22^ and Bracken2 v2.6.0 was used estimate species abundance.^23^ Alpha and beta diversity analyses were performed using the phyloseq v1.27.2 and vegan v2.5-4 packages in R 4.0.2.^24^

### LC-MS/MS-Based Metabolite Profiling Method

A total of 180 to 250 metabolites were measured in plasma samples using mutiple reaction monitoring-based liquid chromatography with tandem mass spectrometry (LC-MS) metabolite profiling techniques as previously described.^25,26^ Briefly, hydrophilic interaction liquid chromatography/positive ion mode MS detection to measure polar metabolites are conducted using an LC-MS system comprised of Agilent 1260 Infinity HPLC coupled to 4000-QTRAP mass spectrometer (Sciex). Plasma samples (10 µL) were prepared via protein precipitation with the addition of nine volumes of 74.9:24.9:0.2 v/v/v acetonitrile/methanol/formic acid containing stable isotope-labeled internal standards (valine-d8, Sigma-Aldrich; St. Louis, MO; and phenylalanine-d8, Cambridge Isotope Laboratories; Andover, MA). The samples were centrifuged (20 min, 15,000 × g, 4°C), and the supernatants were injected directly onto a 150 × 2 mM, 3 µm Atlantis HILIC column (Waters). The column was eluted isocratically at a flow rate of 250 µL/min with 5% mobile phase A (10 mM ammonium formate and 0.1% formic acid in water) for 0.5 minute followed by a linear gradient to 40% mobile phase B (acetonitrile with 0.1% formic acid) over 10 minutes. MS analyses were carried out using electrospray ionization in the positive ion mode using scheduled mutiple reaction monitoring method. Multiquant software (version 3.0.3, Sciex) was used for automatic peak integration followed by manual review of all peaks for quality of integration. Chemists were blinded to study sample assignments, using randomly generated tube numbers. Quality control samples were randomly inserted into sample sequence for quality assurance.

Central metabolites including sugars, sugar phosphates, organic acids, purine, and pyrimidines, were extracted from 30 µL of plasma using acetonitrile and methanol and separated using a 100 × 2.1 mM 3.5-μm Xbridge amide column (Waters). Mobile phase A was 95:5 (v/v) water/acetonitrile, with 20 mM ammonium acetate and 20 mM ammonium hydroxide (pH 9.5). Mobile phase B was acetonitrile. Tandem MS analysis for negative mode detection utilizes a high sensitivity Agilent 6490 QQQ mass spectrometer equipped with an electrospray ionization source. The settings were as follows: sheath gas temperature, 400°C; sheath gas flow, 12 L/min; drying gas temperature, 290°C; drying gas flow, 15 L/min; capillary, 4000 V; nozzle pressure, 30 psi; nozzle voltage, 500 V; and delta EMV, 200 V. Detailed methods have been described previously.^25,26^ Raw data were processed using MassHunter Quantitative Analysis Software (Agilent). Volcano plots were generated using MetaboAnalyst (http://www.metaboanalyst.ca), enabled by R software packages,^27^ with all code packages accessible for free.

### Behavior Tests

The delirium-like phenotype after anesthesia/surgery in mice was identified by using a battery of tests as previously suggested.^28^ Mice in the study had a series of behavioral tests including buried food test, open-field test, novel object test, and finally Y-maze test at 6 and 24 hours after Sham or anesthesia/surgery.

### Novel Object Recognition Test

The test was performed according to published protocol.^29^

### Buried Food Test

The buried food test was performed as described in previous studies with minor modifications.^28^

### Y-Maze and Open-Field Tests

Y-maze and open-field tests are described in online methods, Supplemental Digital Content 1, http://links.lww.com/SLA/E573.

### Bacterial Strains and PCR Confirmation

*C. sporogenes* was purchased from the American Type Culture Collection (#15579). The fldC mutant was a gift from the Sonnenburg lab. Both of them were cultured in beef broth/agar at 37°C in an anaerobic chamber in 5% hydrogen, 10% CO_2_ and 85% N_2_. PCR primer sets were ordered from Invitrogen (CA) designed to produce a ~600-bp product for *C. sporogenes* and ~2800-bp product for the mutant, using the following parameters: 94.0°C for 30 seconds; 60.0°C for 30 seconds; 72°C for 90 seconds, a total of 35 cycles.

### Cell Culture

Mouse hippocampal HT-22 cell line (Millipore, SCC129) was maintained in Dulbecco’s modified Eagle medium/high glucose (Gibco) supplemented with 10% fetal bovine serum and 100 U/mL penicillin/streptomycin (Gibco). Cultures were maintained in a humidified 37°C and 5% CO_2_ incubator. Cells were used at 80% to 85% confluence. IPA was solubilized and diluted to the final concentrations in culture medium. Control group was treated with solvent only.

### Protein Extraction and Western Blotting

Frozen tissue was minced while still lightly frozen and homogenized in RIPA buffer (Cell Signaling, Danvers, MA, USA) with protease inhibitors (Thermo Fisher, Waltham, MA, USA). The samples were then centrifuged at 21,000 g for 10 minutes at 4°C. For immunoblots, protein samples were resolved on 10% sodium dodecyl-sulfate polyacrylamidegel electrophoresis, at a constant voltage of 100 V for 2 hours. The proteins resolved in the gel were then transferred to poly(vinylidene fluoride) membranes. Antibodies: anti-peroxisome proliferator-activated receptor gamma coactivator 1-alpha (PGC-1α) (1:1000, rabbit polyclonal; Abcam, Cambridge, MA, USA), and anti-β-actin (1:5000, mouse monoclonal, Sigma, St. Louis, MO, US).

### Hippocampal Microinjection

A stereotaxic device was used for head fixation under isoflurane anesthesia using pulled glass pipette tip for hippocampal injection. A total of 250 nL of virus (10^12^ vg/L) was injected for each hippocampus.^30^ For DREADD experiment, C21 (HelloBio) was intraperitoneally injected at 1 mg/kg 1 hour before behavioral tests.

### Statistical Analysis

For metabolomic analysis, the *P* value was adjusted for repetitive measurements. One-way analysis of variance was used for multiple group comparisons for the same time point, post-hoc Fisher’s least significant difference test was performed where appropriate. For comparison of 2 groups, t test was used. For metabolomic analysis, the *P* value was adjusted for repetitive measurements.

## RESULTS

### Metabolomic Features of POD in Humans

**FIGURE 1:**
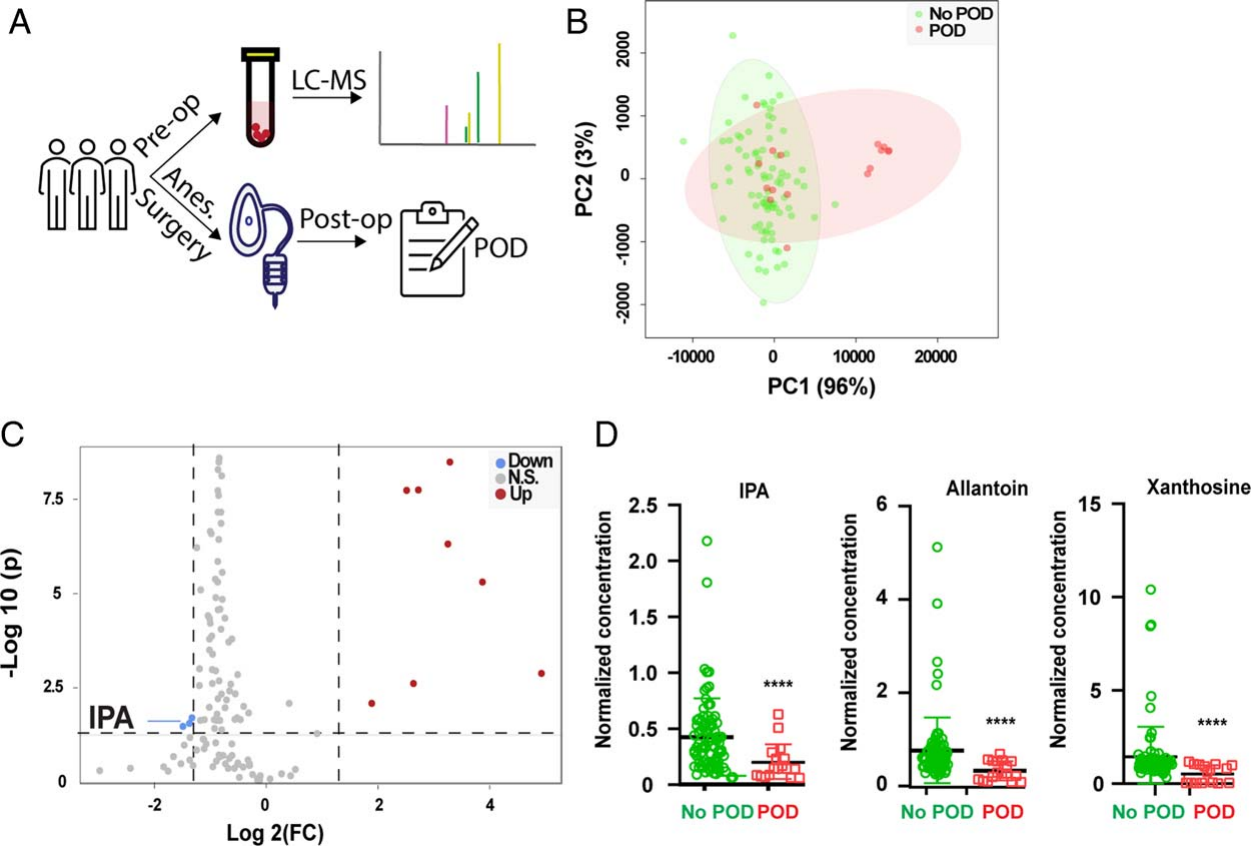
Microbiome derived metabolite, IPA, is implicated in POD. (A) Flowchart for human subjects study. Plasma samples were obtained before anesthesia and surgery (N=103), followed by LC-MS metabolomic examination. All subjects were followed up for the development of POD. Comparisons were made in metabolomics between subjects with POD (N=17) and those without POD (N=86). (B) Principal component (PC) analysis. PC1 and PC2 could separate subjects with POD from those without POD. (C) Volcano plot of all metabolites. X axis: log2 fold-of-change (Log2 FC); Y axis: −log 10 (adjusted *P* value). Cutoff was set using FC 2.5 and adjusted *P* value of 0.05. Blue color represents metabolites that were decreased in POD group, whereas red color represents metabolites that were increased in POD group. (D) Comparison of 3 metabolites identified by volcano plot. ****P<0.0001 with t test, when adjusted for repetitive measurements, all 3 *P* values were less than 0.05. IPA indicates indole-3-propionic acid; LC-MS, liquid chromatography with tandem mass spectrometry; POD, postoperative delirium.

Of the 103 subjects whose blood samples were available for metabolomics study, 17 subjects developed POD, whereas the rest 86 did not. When the demographics of subjects with POD (POD group) and without POD (no-POD group) were examined, age, sex, education background, and type of surgeries were not statistically different between the 2 groups (Table 1). Blood samples were analyzed using LC-MS (Fig. 1A). Principal component analysis showed that the 2 groups could be reasonably separated using 2 principal components (Fig. 1B). A detailed comparison of features between the 2 groups was then carried out by considering both fold-of-change and adjusted *P* value for each metabolite (Fig. 1C). Using 2.5-fold-change and adjusted *P* value of 0.05 as cutoffs, 11 significantly different molecules were identified (Table 2). Among them, the levels of IPA, allantoin, and xanthosine were decreased, whereas the rest 8 were increased in the POD group compared with the no-POD group (Figs. 1C, D). We posited that the molecules with decreased abundance might protect against POD. Simple logistic regression was performed for each of the 3 molecules with decreased abundance: all exhibited significantly negative correlations with POD (odds ratio <1 and *P*<0.05, Supplementary Data Table 1, Supplemental Digital Content 2, http://links.lww.com/SLA/E574). Interestingly, IPA has known neuroprotective properties and is derived from gut microbiota metabolism of tryptophan,^31–34^ suggesting a plausible link between gut microbiota metabolite and POD. When age and Mini-Mental State Exam scores were considered as covariables, the inverse relationship between IPA and POD remained significant (Supplementary Data Table 2, Supplemental Digital Content 2, http://links.lww.com/SLA/E574).

**TABLE 1:** Demographic Characteristics of No-POD and POD Patients

	Level	No-POD	POD	*P*
n		86	17	
Age (median [IQR])		72.00 [69.00, 77.00]	74.00 [70.00, 78.00]	0.544
Sex (%)	F	45 (52.3)	8 (47.1)	0.856
	M	41 (47.7)	9 (52.9)	
Education (%)	Bachelor’s degree	41 (47.6)	10 (58.8)	0.055
	High school graduate, GED	7 (8.1)	0 (0.0)	
	Master’s degree	17 (19.7)	1 (5.9)	
	Some college, associate degree	8 (9.3)	2 (11.8)	
	Doctoral degree	6 (7.0)	0 (0.0)	
	Unknown	7 (8.1)	4 (23.5)	
Type of surgery (%)	LHR	11 (12.7)	2 (11.8)	0.743
	LKR	20 (23.2)	4 (23.5)	
	RHR	15 (17.4)	1 (5.9)	
	RKR	29 (33.7)	8 (47.1)	
	Spine surgery	11 (12.8)	2 (11.8)	
Pre-op_MMSE (median [IQR])		29.00 [29.00, 30.00]	29.00 [28.00, 30.00]	0.421

Descriptive statistics were summarized using means and standard deviations or medians and quantiles (25th and 75th percentiles) for numeric variables according to their data distribution. Categorical variables were reported using frequencies and percentages. Categorical variables were compared using Fisher’s exact test or χ^2^ test for categorical variables (depending on sample size). All statistical analyses were performed using R statistical software V3.6 (The R foundation, Vienna, Austria) and Rstudio V1.2 (Rstudio PBC, Boston, MA). All tests were 2-tailed, *P*<0.05 was considered statistically significant.

F indicates female; LHR, left hip replacement; LKR, left knee replacement; M, male; MMSE, Mini-Mental State Exam; RHR, right hip replacement; RKR, right knee replacement.

**TABLE 2:** Features Identified Using Volcano Plot

	FC	log2(FC)	*P* adjusted
Saccharopine	9.7682	3.2881	3.26E-09
Phosphocreatine	6.6061	2.7238	1.79E-08
Oxalic acid	5.712	2.514	1.83E-08
N-Acetyl-L-Aspartic acid	9.5305	3.2525	4.86E-07
DDHAP/Glyceraldehyde-3-P	14.629	3.8707	4.97E-06
N-Acetyl-L-Glutamic acid	30.419	4.9269	0.001316
Glutathione Disulfide	6.2175	2.6363	0.002445
N-Acetyl-L-Glutamine	3.7046	1.8893	0.008206
Indole-3-propionic acid	0.39818	−1.3285	0.019883
Allantoin	0.38598	−1.3734	0.027868
Xanthosine	0.35625	−1.489	0.03347

Metabolites of subjects with postoperative delirium (POD) and those without POD were compared using cutoff: fold-of-change (FC) 2.5, and adjusted *P* value 0.05 (*P* value adjusted for repetitive measurements). A total of 11 molecules were differentially abundant between the 2 groups: 3 were decreased in the POD group (FC less than 1), whereas 8 were increase in the POD group.

### IPA, A Gut Microbiota Metabolite, Protects Against POD Development

**FIGURE 2:**
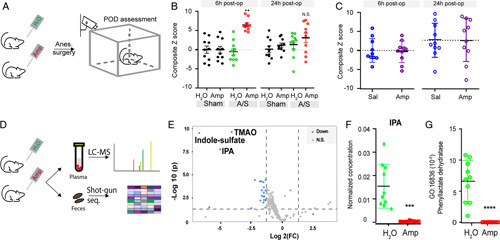
Gut microbiota perturbation exacerbates POD-like behavior. (A, B) Gut microbiota perturbation with oral ampicillin worsened POD-like behavior. (A) Flowchart of mice gut microbiota perturbation followed by anesthesia-surgery (A/S) and POD behavioral testing. (B) Behavior composite Z score at 6- and 24-hour postoperatively. For each time point, comparison of the groups was carried out using 1-way ANOVA, post-hoc LSD test was performed where appropriate. **P<0.01 “H_2_O, A/S” versus “Amp, A/S.” N.S., *P*>0.05 with 1-way ANOVA. (C) Intrathecal administration of ampicillin does not significantly alter POD-like behavior. Mice received daily intrathecal administration of saline (Sal) or Ampicillin (Amp) for 2 weeks followed by anesthesia and surgery (N=10 per group). POD-like behaviors were assessed to compute Z scores. N.S., *P*>0.05, t test. (D–G). Oral ampicillin treatment perturbates gut microbiota and its metabolites. (D) Flowchart for metabolomics and fecal shot-gun sequencing for mice received oral ampicillin treatment. (E) Volcano plot of metabolites that were significantly different between mice received ampicillin versus those received regular water. Cutoff thresholds were the same as in human study. Blue color represents metabolites that were significant decreased after ampicillin treatment, with the top 3 metabolites specified. N=10 each group. (F) IPA level. ***P<0.001 when adjusted for repetitive measurements (t test). (G) An IPA-related GO term, 16836, was decreased by gut microbiota perturbation using oral ampicillin. Transcripts number for GO term 16836 from each animal in both groups were plotted and compared. ****P<0.0001, t test. ANOVA indicates analysis of variance; GO, gene ontology; IPA, indole-3-propionic acid; LSD, Fisher’s least significant difference; POD, postoperative delirium.

**FIGURE 3:**
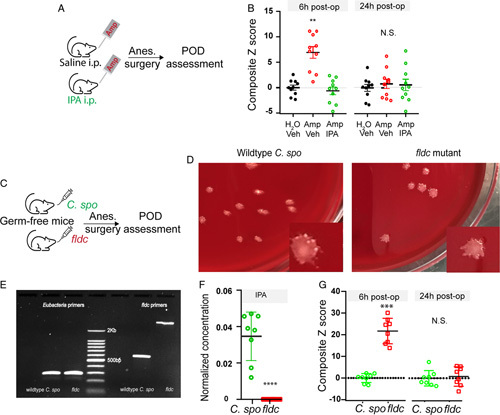
IPA is critical for POD. (A, B) Exogenous IPA administration protects against POD development. (A) Flowchart: mice were fed on water supplemented with ampicillin, and received saline or IPA i.p. injection twice daily for 14 days. After A/S, behavior tests were performed to assessor for POD. (B) Behavior composite Z score at 6- and 24-hour postoperatively. For each time point, comparison of the groups was carried out using 1-way ANOVA, post-hoc LSD test was performed where appropriate. N=10 each group. **P<0.01 “Amp, Vehicle” versus “Amp, IPA”. N.S., *P*>0.05 with 1-way ANOVA, no post-hoc analysis was performed. (C–G) Mutant Δ*fldc* strain promotes POD-like behavior in germ-free mice. Wild-type *Clostridium sporogenes* and mutant Δ*fldc* strain were used to colonized germ-free mice (N=8 each group). Two weeks later, mice underwent anesthesia-surgery followed by behavioral testing. (C) Flowchart of experimental design. (D, E) Confirmation of colonization. Fecal pellets were dissolved in sterile PBS follow by culture (D) or PCR (E). (D) Forty eight to 72 hours after plating, shown representative picture of each group demonstrating one type of colony shape for mice colonized with wild-type *C. sporogenes* or mutant Δ*fldc* strain. E) PCR confirmation of colonization. Fecal DNA was amplified with 2 sets of PCR primers: one for all eubacteria; the other set (*fldc* primers) distinguishes wild-type *C. sporogenes* or mutant *fldc* strain. For the *fldc* primers, wild-type *C. sporogenes* yields a band at ~600 bp, whereas *fldc* mutant yields a band at 2.3 Kb. (F) Plasma IPA concentration from germ-free mice received wild-type *C. sporogenes* or mutant Δ*fldc* strain. ****P<0.0001, t test. (G) Behavior composite Z score at 6- and 24-hour postoperatively. **P<0.01, N.S. *P*>0.05, t test. ANOVA indicates analysis of variance; A/S, anesthesia-surgery; IPA, indole-3-propionic acid; LSD, Fisher’s least significant difference; PCR, polymerase chain reaction; POD, postoperative delirium.

Next, we examined whether gut microbiota perturbation would affect POD development. For this, mice were fed on water supplemented with ampicillin (Amp group) or regular water (H_2_O group) (Fig. 2A). Both groups underwent anesthesia/surgery, followed by postoperative assessment of neurological cognitive function (novel objective recognition; buried food test; Y-maze; and open-field). These behavioral assays have been reported to capture key clinical features of POD,^28,35^ with composite Z score as a quantitative assessment (CAM in mice).^28^ Amp group did worse in those behavioral assays at 6 hours postoperatively than the H_2_O group (Fig. 2B, Supplementary Data Table 3, Supplemental Digital Content 2, http://links.lww.com/SLA/E574). At postoperative 24 hours, the behavioral abnormalities in the Amp group largely resolved (Fig. 2B), suggesting the temporary nature of these deficits, consistent with clinical features of POD. The mice used in the study were 4 to 6 months old adults. In the H_2_O group with “normal” gut microbiota, these mice displayed relative “resilience” against POD, consistent with the clinical observation that young adults patients are less susceptible to POD than older adults.^36^ Notably, the observed behavioral changes were unlikely due to pain or motor weakness induced by surgical trauma, as animals in all groups displayed comparable hindpaw mechanical withdrawal thresholds and total travel distance in the open-field assay, regardless their surgery or antibiotics treatment status (Supplementary Data Figs. 1A, B, Supplemental Digital Content 2, http://links.lww.com/SLA/E574). In addition, we ruled out systemic absorption of ampicillin as the cause of POD-like behavior. Intrathecal daily injection of ampicillin as described^8^ did not alter the development of POD-like behavior (Fig. 2C), suggesting that effects of oral ampicillin were unlikely mediated by its direct systemic absorption, although several antibiotics have been reported to have neuro modulatory effects.^37^ We performed metabolomic and shot-gun metagenomic studies in blood and feces samples, respectively, to examine ampicillin-induced changes (Fig. 2D). Using the same cutoffs as the human metabolomics study, the top 3 molecules that were decreased in Amp group were IPA, trimethylamine N-oxide, and indole-3 sulfate, all of which are gut microbiota-derived molecules^32,38^ (Fig. 2E). Concordant with robust metabolomic changes, shot-gun sequencing revealed community structure changes as well as changes at the phylum and species levels (Supplementary Data Figs. 2A–C, Supplemental Digital Content 2, http://links.lww.com/SLA/E574). *C. Sporogenes* and *Peptostreptococcu*s species have been demonstrated to produce IPA.^31,39^ When the shot-gun metagenomics sequencing reads of *C. Sporogenes* and *Peptostreptococcu*s species were mapped in taxonomy, these IPA-producing bacteria were eliminated in the Amp group (Supplementary Data Figs. 2D, E, Supplemental Digital Content 2, http://links.lww.com/SLA/E574), consistent with the dramatically decreased levels of IPA in the Amp group (Fig. 2F). It has been shown that generation of indole family members by gut microbiota are related to the *fldABC* gene family.^33,39^ The *fldABC* genes, encoding 2-hydroxyacyl-CoA dehydratases, belong to the GO term 0016836 (hydro-lyase activity). Interestingly, genes from this GO were dramatically decreased in the Amp group, as revealed by shot-gun sequencing (Fig. 2G), in line with the elimination of *C. Sporogenes* and *Peptostreptococcu*s species. As such, perturbation of gut microbiota using oral ampicillin introduced robust metabolomic changes, including decreased IPA levels, and promoted POD-like behavior. To directly assess IPA in POD, IPA was administered to mice that received ampicillin who would otherwise be prone to developing POD-like behavior (Fig. 3A). Exogenous administration of IPA significantly deterred the development of POD-like behavior, as animals received oral ampicillin and IPA performed significantly better than mice received ampicillin and saline (Fig. 3B and Supplementary Data Table 3, Supplemental Digital Content 2, http://links.lww.com/SLA/E574), supporting a protective role of IPA against the development of POD-like behavior.

### Genetically Modified Bacterium that Does not Produce IPA Promotes POD-Like Behavior

To determine if lack of IPA could precipitate POD-like behavior, we colonized germ-free mice with wild-type *C. Sporogenes* or a mutant strain Δ*fldC* that does not produce IPA^33^ (Fig. 3C). Successful colonization was confirmed by fecal sample culture yielding colony shapes that were uniform with rough edges, as well as PCR using a set of primers that differentiated wild-type *C. Sporogenes* and Δ*fldC* (Figs. 3D, E).^33^ In addition, IPA was nearly absent in mice colonized with Δ*fldC* but abundantly present in mice colonized with wild-type *C. Sporogenes* (Fig. 3F). After anesthesia/surgery, animals that were colonized with Δ*fldC* did worse in behavioral tasks when compared with those that received wild-type *C. Sporogenes* (Fig. 3G, Supplementary Data Table 3, Supplemental Digital Content 2, http://links.lww.com/SLA/E574), suggesting lack of IPA could precipitate the development of POD-like behavior.

### Hippocampal Interneuron PGC-1α Overexpression Protects Against POD Development Linked to Gut Microbiota Perturbation

**FIGURE 4:**
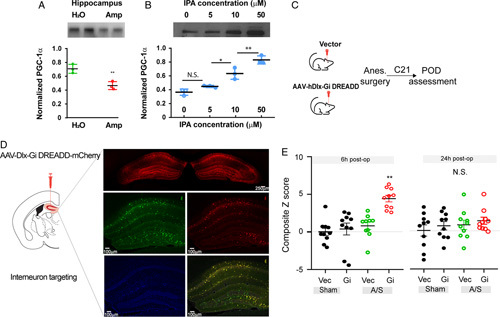
PGC-1α and GABAergic interneurons in POD. (A) Decreased PGC-1α levels in mice received oral ampicillin. Hippocampal PGC-1α levels were compared between mice received regular water versus water supplemented with ampicillin (N=4), **P<0.01, t test. (B) Exogenous IPA treatment increased PGC-1α in HT-22 cells. Six hours after IPA treatment at indicated concentrations, PGC-1α levels were determined (N=3 for each concentration, 1-way ANOVA followed by post-hoc tests, N.S., *P*>0.05, *P<0.05, **P<0.01). (C–E) Chemogenetic inhibition of hippocampal GABAergic interneurons promotes POD-like behavior. Mice received AAV-dDlx-Gi DREADD-dTomato or AAV-Dlx-dTomato vector control then underwent anesthesia-surgery (N=10 group). (C) Flowchart of experimental design. (D) GABAergic interneuron targeting. Green: anti-GAD67-FITC;Red: dTomato; Blue: DAPI. Scale bar: 100 μm unless otherwise specified. The specificity of interneuron target was assessed by dTomato and anti-GAD67 double-positive cells among GAD67-positive cells. (E) Behavior composite Z score at 6- and 24-hour postoperatively. For each time point, comparison of the groups was carried out using 1-way ANOVA, post-hoc LSD test was performed. **P<0.01 “Gi, A/S” versus “Vec, A/S.” N.S., *P*>0.05 with 1-way ANOVA indicates ANOVA, analysis of variance; IPA, indole-3-propionic acid; LSD, Fisher’s least significant difference; PGC1α, peroxisome proliferator-activated receptor gamma coactivator 1-alpha; POD, postoperative delirium.

**FIGURE 5:**
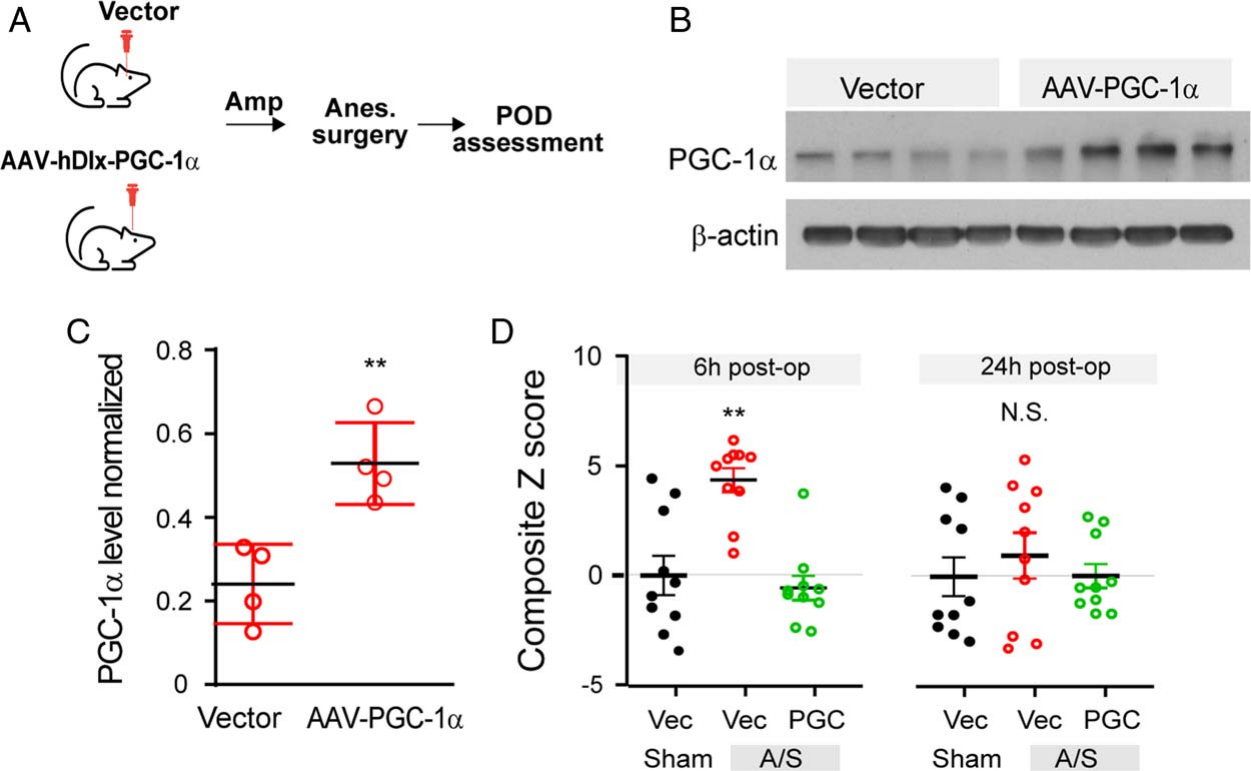
Hippocampal interneuron overexpression PGC-1α protect against POD. (A–D) Hippocampal interneuron overexpression of PGC-1α protects against development of POD-like behavior. Hippocampal microinjection of AAV-dDlx-PGC-1a-2A-mCherry or AAV-hDlx-mCherry vector control (N=10 each group). (A) Flowchart of experimental design. (B, C) Hippocampal PGC-1α levels. Mice were sacrificed after behavioral testing and hippocampal levels were examined PGC-1α using Western blot, using β actin as internal control. **P<0.01, t test. (D) Behavior composite Z score at 6- and 24-hour postoperatively. For each time point, comparison of the groups was carried out using 1-way ANOVA, post-hoc LSD test was performed where appropriate. **P<0.01 “PGC, A/S” versus “Vec, A/S”. N.S., *P*>0.05 with 1-way ANOVA. ANOVA indicates analysis of variance; LSD, Fisher’s least significant difference; PGC1α, peroxisome proliferator-activated receptor gamma coactivator 1-alpha; POD, postoperative delirium.

Mitochondria dysfunction is one of the key pathogenic factors implicated in postoperative cognitive disorder and POD.^3,4^ PGC-1α serves as a master regulator of mitochondria biogenesis that plays a key role in neuronal function,^40,41^ including the functional integrity of GABAergic interneurons.^42–44^ We compared animals received regular water versus those who received water supplemented with oral ampicillin, and found the latter with lower levels of PGC-1α in the hippocampus (Fig. 4A), a critical region implicated in learning, memory, and cognition. We examined mice that were fed on water supplemented with ampicillin, and compared those also received IPA injection versus saline control. Results showed that IPA administration increased hippocampal PGC-1α expression (Supplementary Data Fig. 3, Supplemental Digital Content 2, http://links.lww.com/SLA/E574). When a hippocampal HT-22 cell line was treated with IPA or vehicle control, there were dose-dependent increases of PGC-1α expression (Fig. 4B). As PGC-1α is critical for the functional integrity of interneurons, including parvalbumin interneurons,^42,43^ we asked if interneuron dysfunction could promote the development of POD-like behavior (Fig. 4C). We used Designer Receptors Exclusively Activated by Designer Drugs (DREADD)–based chemogenetic tools^45^ to inhibit interneurons. Specifically, we used adeno-associated virus (AAV)-mediated expression of an inhibitory DREADD:AAV-hDlx-hM4D (Gi DREADD)-dTomato,^46^ a recently developed tool for efficient interneuron targeting. AAV-hDlx-Gi DREADD-dTomato was able to target about 80% all Gad67+ interneurons in the hippocampus (Fig. 4D), consistent with previous reports.^46^ We found that interneuron inhibition by activating Gi DREADD with C21^47^ promoted POD-like behavior (Fig. 4E, Supplementary Data Table 3, Supplemental Digital Content 2, http://links.lww.com/SLA/E574), supporting a key role of interneurons in the development of POD. We then asked if PGC-1α overexpression in hippocampal interneurons could ameliorate POD susceptibility in animals receiving oral ampicillin (Fig. 5A). For this, we constructed AAV-hDlx- PGC-1α -2A- mCherry for PGC-1α overexpression (Figs. 5B, C). In animals that received oral ampicillin who would be prone to POD development, we found those that were pretreated by hippocampal injection of AAV-hDlx- PGC-1α -2A-mCherry exhibited better behavioral indices than those that were pretreated by AAV-hDlx- mCherry vector control (Fig. 5D, Supplementary Data Table 3, Supplemental Digital Content 2, http://links.lww.com/SLA/E574), consistent with a protective role of PGC-1α in POD. As such, hippocampal interneuron PGC-1α was implicated in POD protection.

## DISCUSSION

We took advantage of a metabolomic approach to screen human plasma samples that led to the discovery of several candidate molecules that were linked to POD. Among these molecules, IPA is derived from the gut microbiota. We then validated findings in humans using a preclinical animal model for POD. Previous research has identified age and preexisting baseline cognitive deficits as risk factors for POD. As nonmodifiable factors, they are not amenable to intervention in the perioperative setting. On the other hand, the gut microbiota and its metabolites are modifiable factors that are modulated by diets and medications.^48^ As such, the identification of a gut microbiota-derived molecule, IPA, in the pathogenesis of POD represents a unique opportunity for POD prevention and treatment.

Indoles are the main source of fecal smell. At low concentrations, however, they are widely used in perfumes. As a indole family member, IPA has previously been shown to have antioxidant properties with significant neuroprotection against amyloid beta in cultured neurons.^34^ In addition, in a rodent forebrain ischemia model, IPA protected the hippocampal CA-1 area neurons from ischemic damage.^49^ More recently, it has been shown that intermittent fasting promotes axonal regeneration after sciatic nerve crush injury in mice, and that this beneficial effect is linked to IPA.^50^ As such, our results that IPA protects against the development of POD are in line with a growing body of evidence supporting IPA’s neuroprotective role. Notably, indole family members exert heterogeneous neurological functions. For example, indole sulfate has been shown to promote neuroinflammation and neurodegeneration.^51^

Gut microbiota has been implicated in many neurological conditions, such as autism, multiple sclerosis, AD, and neuropathic pain.^8,52,53^ More recently, gut microbiota has been implicated in POD.^11,54^ For example, lactobacillus was associated with aging-associated development of POD,^10^ raising an intriguing possibility for therapeutics development using gut microbiota. However, we must note that it is difficult to define what constitutes a “normal” gut microbiota, and that bacteria carried in “healthy” volunteers might become pathogenic in others. For example, extended-spectrum beta-lactamase–producing *Escherichia coli* from a presumably asymptomatic “healthy” donor was linked to *E. coli* bacteremia after fecal microbiome transplantation and in one case, patient death.^55^ Small molecules, including those derived from gut microbiota, have defined development pathways for pharmacokinetics and pharmacodynamics assessment as well as safety profiling.

We found that PGC-1α, a master regulator of mitochondria biogenesis, was implicated in IPA’s role for POD. Decreased hippocampal PGC-1α expression coincided with POD susceptibility in mice received oral ampicillin. Overexpression of PGC-1α in hippocampal interneurons prevented POD development in otherwise susceptible animals. Hippocampal interneurons, particularly cholecystokinin expressing interneurons in the dentate gyrus, were critical for memory generalization and discrimination.^56^ PGC-1α knockout mice displayed significant neurological symptoms, including hyperactivity and neurodegeneration.^57^ They also tended to perform poorly in the hippocampus-based spatial learning paradigm.^58^ Importantly, interneuron-specific PGC-1α knockout mice through transgenic expression of Cre recombinase under the control of a Dlx5/6 promoter displayed mania-like behavior in a battery of tests including spontaneous activity, elevated plus maze, forced swim test, and tail suspension test.^59^ In humans, delirium often presents with symptoms that are suggestive of disinhibition, such as agitation, hallucination, and delusion.^60^

This study has some limitations. First, for the human study, we only collected data on postoperative days 1 and 2, as these patients were typically discharged from hospital at day 3. Second, Rodent models do not completely recapitulate all clinical features of POD in humans.^61,62^ Several different rodent models have been used to study POD, including the Z score system that we used in this study,^28^ and 5-choice serial reaction time task for inattention aspect of delirium.^63^ For the Z score system, a battery of assessment tools were employed to examine natural behavior (buried food and an open-field test) to probe for attention and awareness as well as learned behavior (Y-maze test, novel object recognition test) to assess cognition and memory. These behaviors (natural and learned) are presumably dependent on consciousness, attention, awareness, and organized thinking. When conducted in series, these test could capture acute onset and fluctuation of POD-like behavior. In our study, the increases in Z score reflected changes in behaviors in buried food, novel object recognition, Y-maze, and open-field test, with no single behavior being the primary driving force. As such, these abnormalities suggest an overall brain dysfunction. Delirium in humans involves multiple domains, such as memory, orientation, language, visuospatial ability, and perception, reflecting global brain dysfunction.^64^ Notably, our detection of POD-like behavior at 6 hour postoperatively was different from the usual peak of POD in humans at postoperative day 1 to 2. This “discrepancy” was probably related to different life spans of rodents and humans, as well as their differences in POD behavior. Third, interneuron targeting using Dlx5/6 promoter has been widely used and has relatively high specificity.^46^ However, a very small percentage of excitatory neurons could have been nonspecifically targeted. Despite these limitations, the human study and mouse study were concordant in supporting a role for gut microbiota metabolite in POD. It is also noteworthy that many mechanisms have been implicated in the pathogenesis of POD including mitochondria dysfunction, neuroinflammation, etc.^3,4,65^ It is interesting to speculate that gut microbiota might be associated with some of these known mechanisms. For example, our results suggest PGC-1α, a known regulator for mitochondria biogenesis, was modulated by gut microbiota.

Taken together, we found that IPA, a gut microbiota metabolite, was inversely correlated with POD development in humans. In preclinical animal models for POD, lack of IPA promoted, whereas exogenous administration of IPA deterred POD-like behavior. This protective role of IPA was mediated, in part, by PGC-1α in hippocampal interneurons. Our results suggest that harnessing the gut microbiota and its metabolites could facilitate developing preventive and therapeutic strategies against POD.

## Supplementary Material
