# Supplementary material for: Indole-3-Propionic Acid, a Gut Microbiota Metabolite, Protects Against the Development of Postoperative Delirium
Source: Ann Surg. 2023 Apr 27;278(6):e1164–74. doi: 10.1097/SLA.0000000000005886 (PMC10603211; doi:10.1097/SLA.0000000000005886)
Supplement: Supplementary file 1 [file sla-278-e1164-s001.docx]

**Novel object recognition test.** It was carried out in two sessions divided by a 10-minute intersession intervals in an open-field plastic apparatus (33×33×20 cm). During the first session, the animal was free to explore two similar objects, and during the second session (test session), one of the objects was replaced by an unfamiliar, novel object. For both sections, 20-s exploration of both objects or a 10-min period was set as experimental period.

**Buried food test.** We provided each mouse 2 pieces of the sweetened chow pellet (1 centimeter long) two days before the test. One hour before test, mice in home cages were placed in the testing room for habituation. We prepared the test cage with clean bedding (3cm thick). During tests, one sweetened chow pellet (0.5 centimeter) was placed below the surface of bedding in a random fashion, which it was not visible. The mouse was put in the center of the test cage and we manually recorded the latency of the mouse to uncover and eat the food. Latency was defined as the time from when the mouse was placed in the cage until when the mouse found the food pellet and grasped it in the forepaws and/or teeth.

**Y-maze Test.** The Y-maze test included a training session and a testing session, which were separated by an inter-trial interval of two hours. The Y-maze apparatus consisted of three arms (A, B and C) with an angle of 120 degrees between each arm. Arm A and B were always open during the training and testing sessions, while the arm C designated as the novel arm was closed during training session and opened during testing session. The training session was 10 minutes in duration. For testing session, the mouse was placed back in the Y-maze in the same start arm with free access to all three arms for five minutes. The time spent in and the number of entries into the novel arm were recorded.

**Open Field Test.** The apparatus was a square chamber (57×57×57cm), which was divided into the center area (40×40cm) and the peripheral area. For testing, mice were gently placed in the center area and was allowed to move freely for 5 minutes. A Video camera connected to the Any-Maze animal tracking system software (Stoelting Co., Wood Dale, IL) was used for tracking. The total move distance (centimeters), the time (seconds) spent in the center area, the latency (seconds) to the center area of the open field, and the freezing time (seconds) were recorded and analyzed.
