# Supplementary material for: Indole-3-Propionic Acid, a Gut Microbiota Metabolite, Protects Against the Development of Postoperative Delirium
Source: Ann Surg. 2023 Apr 27;278(6):e1164–74. doi: 10.1097/SLA.0000000000005886 (PMC10603211; doi:10.1097/SLA.0000000000005886)
Supplement: Supplementary file 2 [file sla-278-e1164-s002.docx]

**Supplementary data table 1. Logistic regression model odds ratio (95% confidence intervals) using postoperative outcome as primary outcome.**

|  | OR (95% CI) | p-value |
| --- | --- | --- |
| Indole-3-propionic acid | 0.003 [0.000, 0.115] | 0.006 |
| Allantoin | 0.001 [0.000, 0.023] | 0.001 |
| Xanthosine | 0.017 [0.002, 0.095] | 0.001 |

Logistic regression was used to model POD as primary outcome, using individual metabolites as variables to compute odds ratio.

**Supplementary data table 2. Multiple logistic regression considering IPA levels, age, and MMSE using postoperative delirium as a primary outcome.**

| Variable | OR (95% CI) | p-value |
| --- | --- | --- |
| Age | 1.003 [0.902-1.115] | 0.963 |
| MMSE | 0.879 [0.523-1.477] | 0.627 |
| Indole-3 propionic acid | 0.004 [0.001-0.212] | 0.007 |

Multiple logistic regression was used to model POD as a primary outcome, using individual age, MMSE, and IPA as variables to compute odds ratio.

**Supplementary data table 3. Summary of all behavioral tests**

| ***Gut microbiota perturbation promotes POD-like behavior* (Figure 2B)**  ‘A/S+Amp’, compared to ‘Sham+H_2_O’, ‘Sham+Amp’ or ‘A/S+H_2_O’ (p<0.05) | | |
| --- | --- | --- |
| Parameters | 6Hr | 24Hr |
| **Buried food test** | | |
| Latency to eat food (second) | **↑** | — |
| **Novel object recognition test** | | |
| Novel object time (second) | ↓ | ↓ |
| Time to reach the criterion (second) | **↑** | — |
| **Y maze** |  |  |
| Novel arm entries | — | — |
| Duration in novel arm (second) | ↓ | ↓ |
| **Open field test** |  |  |
| Latency to the center (second) | — | — |
| Time in the center (second) | ↓ | — |
| Freezing time (second) | — | — |
| ***IPA administration alleviates POD* (Figure 3B)**  Group ‘Amp +Vec’, compared to ‘Amp +IPA’ or ‘H_2_O+Vec’(p<0.05) | | |
| Parameters | 6Hr | 24Hr |
| **Buried food test** |  |  |
| Latency to eat food (second) | **↑** | — |
| **Novel object recognition test** |  |  |
| Novel object time (second) | ↓ | ↓ |
| Time to reach the criterion (second) | **↑** | — |
| **Y maze** |  |  |
| Novel arm entries | — | — |
| Duration in novel arm (second) | ↓ | ↓ |
| **Open field test** |  |  |
| Latency to the center (second) | — | — |
| Time in the center (second) | ↓ | — |
| Freezing time (second) | — | — |
| ***Germ-free mice colonized with fldC mutant develops POD-like behavior* (Figure 3G)**  ‘*fldC’* compared to‘wildtype *C.spo’* (p<0.05) | | |
| Parameters | 6Hr | 24Hr |
| **Buried food test** |  |  |
| Latency to eat food (second) | **↑** | — |
| **Novel object recognition test** |  |  |
| Novel object time (second) | ↓ | — |
| Time to reach the criterion (second)  **Open field test**  Latency to the center (second)  Time in the center (second)  Freezing time (second) | **↑**  —  ↓  — | —  —  —  — |
| ***Hippocampal interneuron inhibition worsens POD-like behavior* (Figure 4E)**  ‘A/S+Gi’, compared to ‘A/S+Vec’, ‘Sham+Gi’, or ‘Sham+Vec’ (p＜0.05) | | |
| Parameters | 6Hr | 24Hr |
| **Buried food test** |  |  |
| Latency to eat food (second) | **↑** | — |
| **Novel object recognition test** |  |  |
| Novel object time (second) | ↓ | ↓ |
| Time to reach the criterion (second) | **↑** | — |
| **Y maze** |  |  |
| Novel arm entries | ↓ | — |
| Duration in novel arm (second) | ↓ | ↓ |
| **Open field test** |  |  |
| Latency to the center (second) | **↑** | — |
| Time in the center (second) | — | — |
| Freezing time (second) | ↓ | — |
| ***Hippocampal overexpression of PGC1-α alleviates POD-like behavior* (Figure 5D)**  ‘A/S + Vec’, compared with ‘A/S + PGC-1α’ or ‘Sham + Vec’(p＜0.05) | | |
| Parameters | 6Hr | 24Hr |
| **Buried food test** | | |
| Latency to eat food (second) | **↑** | — |
| **Novel object recognition test** | | |
| Novel object time (second) | ↓ | ↓ |
| Time to reach the criterion (second) | **↑** | — |
| **Y maze** |  |  |
| Novel arm entries | ↓ | — |
| Duration in novel arm (second) | ↓ | — |
| **Open field test** |  |  |
| Latency to the center (second) | **↑** | — |
| Time in the center (second) | — | — |
| Freezing time (second) | ↓ | — |

***Gut microbiota perturbation with oral ampicillin worsens POD-like behavior***. Animals were fed on regular water (H_2_O) or water supplemented with ampicillin (Amp) for two weeks followed by anesthesia-surgery (A/S) or Sham treatment (N=10 each group). Behavioral testing was performed both before A/S and at indicated time points postoperatively.

***IPA administration alleviates POD-like behavior***. Mice were fed on regular water (H_2_O) or water supplemented with ampicillin (Amp), with twice daily intraperitoneal injection of IPA or saline (Vec), for two weeks (N=10 mice each group). All animals underwent anesthesia-surgery, followed by behavioral testing.

***Germ-free mice colonized with fldC mutant develop POD-like behavior***. Germ-free mice were colonized via oral gavage using either wildtype *C. Spo* or mutant *fldc* strain (N=8 mice each group). Two weeks post colonization, mice underwent anesthesia-surgery followed by behavioral testing.

***Chemogenetic inhibition of hippocampal GABAergic interneuron promotes POD-like behavior***. Hippocampal microinjection of AAV-hDlx-Gi-DREADD-dTomato or AAV-hDlx-dTomato vector control were performed in mice. Four weeks were allowed for virus expression, followed by anesthesia-surgery and POD behavior testing (N=10 each group).

***Hippocampal overexpression of PGC1-α alleviates POD-like behavior***. Hippocampal microinjection of AAV-hDlx-PGC-1α-2A-mCherry or AAV-hDlx-mCherry vector control. Four weeks after injection mice were started on water supplemented with ampicillin for two weeks, followed by anesthesia-surgery (A/S) and POD behavior testing (N=10 each group).

For each time point of all experiments, comparison of the groups was carried out using one-way ANOVA.

**Supplementary data figure 1. Total travel distance in open field.**


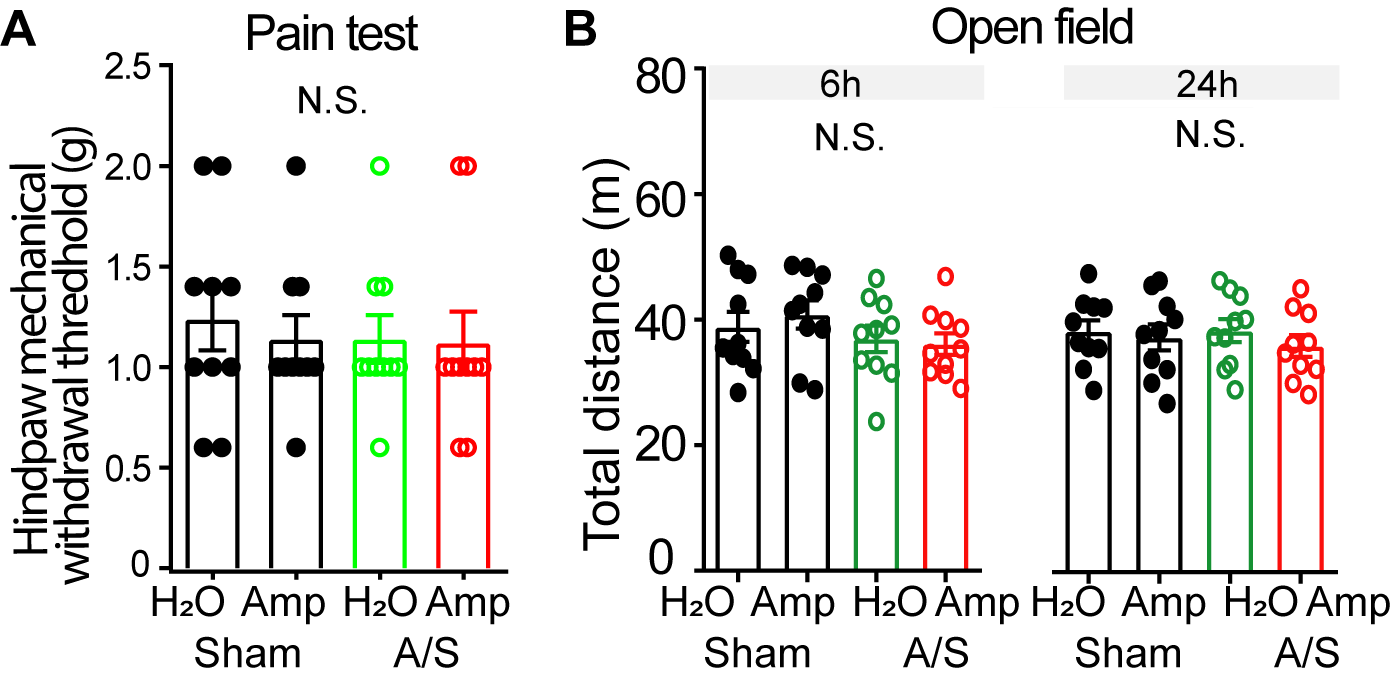


Mice received regular water (H_2_O) and water supplemented with ampicillin (Amp) then underwent either Sham or Anesthesia/Surgery (A/S) followed by hindpaw mechanical withdrawal threshold testing and open field assay (N=10 per group). A) Hindpaw mechanical withdrawal threshold. B) Total travel distance of each animal was tracked and comparisons were made among all groups at 6 and 24 hour postoperatively. There was no statistically significant difference among the groups (N.S., p>0.05, One-way ANOVA).

**Supplementary data figure 2. Gut microbiota changes revealed by shot-gun sequencing.**


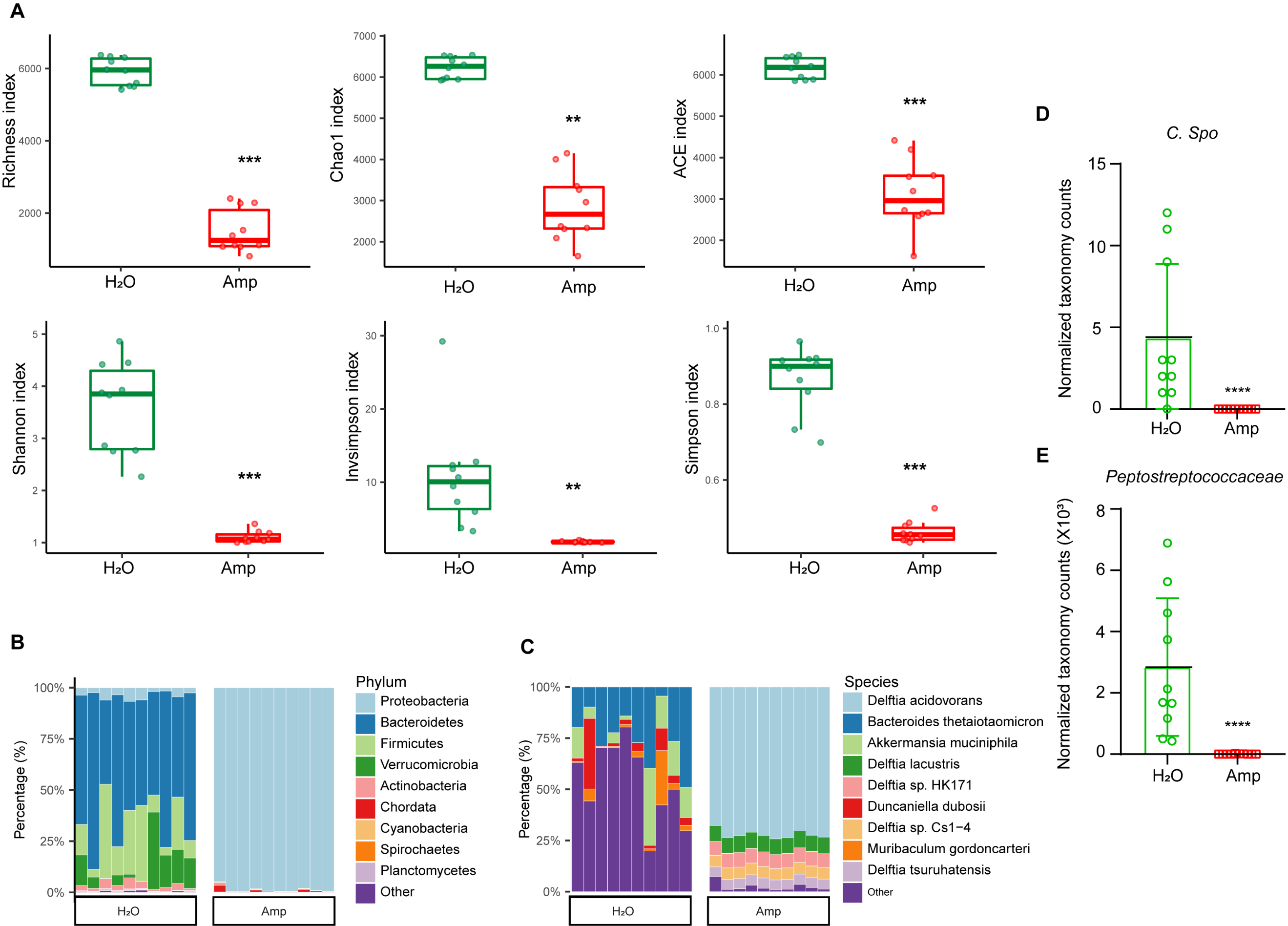


A-E) Oral ampicillin alters gut microbiota composition. Animals were fed on regular water (H_2_O) or water supplemented with ampicillin (Amp) for two weeks followed fecal sample DNA shot gun sequencing. **A**) Bacterial diversity as measured by different indices. **p<0.01, ***p<0.001, t test. **B-C**) Community structure at the phylum and Species levels. **D**) Normalized taxonomy counts of C. Spo, **** p<0.0001, t test. **E**) Normalized taxonomy counts of *Peptostreptococcaceae*. **** p<0.0001, t test

**Supplementary data figure 3. IPA treatment increases hippocampal PGC-1α expression.**


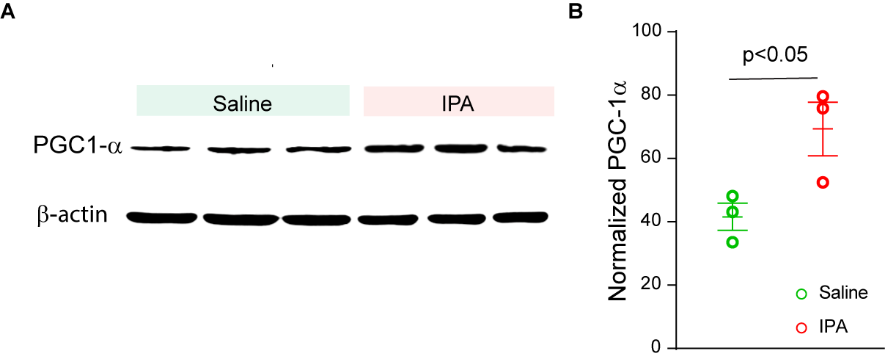


Mice were fed on water supplemented with ampicillin, and received saline (n=3) or IPA (n=3) i.p. injection twice daily for 14 days. Hippocampal PGC-1α was assessed with western blots. A) Western blots. B) Normalized levels of PGC-1α. Statistical analysis: t test.
